# Lymphatic endothelial cells promote productive and latent HIV infection in resting CD4+ T cells

**DOI:** 10.1186/s12985-018-1068-6

**Published:** 2018-10-03

**Authors:** Meghan Schilthuis, Seth Verkaik, Mackenzie Walhof, Andrew Philipose, Olivia Harlow, Derrick Kamp, Bo Ram Kim, Anding Shen

**Affiliations:** Department of Biology, Calvin College, 1726 Knollcrest Circle SE, Grand Rapids, MI 49546 USA

**Keywords:** HIV, Resting CD4+ T, Latent infection, Lymphatic endothelial cells, Viral reservoir

## Abstract

**Background:**

An HIV cure has not yet been achieved because latent viral reservoirs persist, particularly in resting CD4+ T lymphocytes. In vitro*,* it is difficult to infect resting CD4+ T cells with HIV-1, but infections readily occur in vivo. Endothelial cells (EC) line the lymphatic vessels in the lymphoid tissues and regularly interact with resting CD4+ T cells in vivo. Others and we have shown that EC promoted productive and latent HIV infection of resting CD4+ T cells. However, the EC used in previous studies were from human umbilical cords (HUVEC), which are macrovascular; whereas EC residing in the lymphoid tissues are microvascular.

**Methods:**

In this study, we investigated the effects of microvascular EC stimulation of resting CD4+ T cells in establishing viral infection and latency. Human resting and activated CD4+ T cells were cultured alone or with endothelial cells and infected with a pseudotyped virus. Infection levels, indicated by green fluorescent protein expression, were measured with flow cytometry and data was analyzed using Flowing Software and Excel.

**Results:**

We confirmed that EC from lymphatic tissue (LEC) were able to promote HIV infection and latency formation in resting CD4+ T cells while keeping them in resting phenotype, and that IL-6 was involved in LEC stimulation of CD4+ T cells. However, there are some differences between stimulation by LEC and HUVEC. Unlike HUVEC stimulation, we demonstrated that LEC stimulation of resting memory T cells does not depend on major histocompatibility complex class II (MHC II) interactions with T cell receptors (TCR) and that CD2-CD58 interactions were not involved in LEC stimulation of resting T cells. LEC also secreted lower levels of IL-6 than HUVEC. We also found that LEC stimulation increases HIV infection rates in activated CD4+ T cells.

**Conclusions:**

While differences in T cell stimulation between lymphatic EC and HUVEC were observed, we confirmed that similar to macrovascular EC stimulation, microvascular EC stimulation promotes direct HIV infection and latency formation in resting CD4+ T cells without T cell activation. LEC stimulation also increased infection rates in activated CD4+ T cells. Additionally, the present study established a physiologically more relevant model of EC interactions with resting CD4+ T cells and further highlighted the importance of investigating the roles of EC in HIV infection and latency in both resting and activated CD4+ T cells.

## Background

Antiretroviral therapy (ART) is able to control the replication of human immunodeficiency virus type 1 (HIV-1) in patients but cannot eradicate the virus. As a result, HIV persists and HIV patients require lifelong therapy to suppress viremia. A major barrier to eradication is the presence of latent cellular reservoirs, particularly in resting CD4+ memory T cells. Because of the long life span of these cells, their proliferative capacity, and the extremely slow decay rate of the reservoir, this poses the biggest obstacle for an HIV cure. Since the discovery of this reservoir in the 1990s, much has been learned about latency maintenance (reviewed in [1] and [2]), latency reversing agents (reviewed in [3]), and strategies to eliminate the reservoir (reviewed in [4] and [5]). Although much effort has been made to reactivate latent HIV in order to eliminate the reservoir, significant hurdles to this approach have been encountered because cell death did not ensue after latency reversal (reviewed in [6]). Recent innovations involve using latency strengthening agents or CRISPR based systems to further lock latent virus inside the infected cells in order to prevent reactivation [7]. This approach may be promising, but most of the work is still at the in vitro stage. In the meantime, our understanding of how the latent reservoir is established is still very limited.

Based primarily on in vitro evidence, it is believed that HIV can only replicate in activated CD4+ T cells [8–12]. In resting T cells, the virus can enter the cell but either cannot complete reverse transcription [11] or can complete reverse transcription at a much lower efficiency but cannot integrate its cDNA into the host genome [13, 14]. The favored model of latent reservoir formation is that HIV cannot directly infect resting CD4+ T cells. Rather, activated CD4+ T cells are infected and then revert to a resting phenotype with integrated provirus to form the latent reservoir. Various mechanisms were proposed and demonstrated to establish latent infection in these deactivating CD4+ T cells (reviewed in [15]). A recent in vitro study provided evidence that CD4+ T cells undergoing effector-to-memory (activated-to-resting) transition allowed viral integration but down-regulated gene transcription to favor latency formation [16]. This is the traditional model to explain infection of resting CD4+ T cells by HIV and latency formation, but it is not the only model, nor an exclusive model.

A newer model is gaining support in recent years based on in vivo and ex vivo studies which have shown that resting CD4+ T cells were productively infected in vivo, or can be infected directly ex vivo [17–22]. One of the studies found that resting CD4+ T cells support HIV replication in lymphoid tissue (tonsil) explants, whereas purified tonsillar resting CD4+ T cells did not support HIV replication [23]. Another study by Chavez et al. demonstrated that latent infection could be achieved via direct infection of both activated and resting CD4+ T cells, with resting cells displaying a higher propensity for latent as opposed to productive infection [24]. They also found that CD4+ T cells isolated from splenic and tonsillar lymphoid tissues had significantly higher latent infection rates when compared to purified CD4+ T cells isolated from peripheral blood, highlighting the importance of the lymphoid environment in the establishment of HIV latency. In addition, stimulation by cytokines, chemokines, dendritic cells, or stromal fibroblasts can render resting CD4+ T cells permissive for HIV infection and/or latency formation [25–28]. This newer model advocates for direct infection of resting CD4+ T cells, especially in the context of lymphoid tissue or cytokine/chemokine interactions. The two models for the formation of latent reservoir in resting CD4+ T cells are not mutually exclusive, and they may both occur in an HIV+ patient. However, in vivo, T cells do not live alone; they are always surrounded by soluble factors and other cell types. Therefore, it is extremely important to further investigate the lymphoid tissue microenvironment and cell-to-cell interactions within those microenvironments for their role in inducing HIV infection and latency.

Two studies by Choi et al. [29, 30] first showed that stimulation by endothelial cells (EC) rendered resting CD4+ T cells permissive for HIV replication while continuing to exhibit a resting phenotype. EC line the lymphatic vessels in the lymphoid tissues and have constant interactions with T cells trafficking through them. This was the first indication that EC, which physiologically serve as antigen-presenting cells to T cells, particularly in lymphoid microenvironments, might play a significant role in infection of resting CD4+ T cells in vivo*.* In our 2013 study, we verified the findings that upon EC stimulation, resting CD4+ T cells can be productively infected by HIV while remaining in a resting phenotype [31]. We further demonstrated that EC stimulation can result in latent infection in resting CD4+ T cells. Initially, it was thought that stimulations by EC required cell-cell contact and were dependent upon MHC class II - TCR interactions and interactions between CD58, an adhesion molecule expressed by EC and CD2, an adhesion/co-stimulatory molecule expressed by T cells [29, 30]. In our 2017 study, we demonstrated that soluble factors secreted by EC can promote both productive and latent infection of resting CD4+ T cells, though not to the same level as stimulation by cell-cell contact [32]. We also identified IL-6 to be a key soluble factor involved in EC stimulation of resting CD4+ T cells.

From the above-mentioned studies, we have demonstrated the importance of EC in HIV infection and latency formation in resting CD4+ T cells. However, the EC used in the Choi studies and in our own studies were from human umbilical cords (HUVEC). They are considered macrovascular EC, whereas the EC that line the lymphatic vessels in the lymph nodes are microvascular EC. Phenotypical and physiological differences between macrovascular and microvascular EC have previously been observed, even within a single human organ [33]. It has been demonstrated that microvascular EC show lower adherence to other normal cell types [34] and cancer cells [35], respond more strongly to certain growth factors [36], and respond to IL-1 and lipopolysaccharides with higher sensitivity resulting in different chemokine production [37] compared to macrovascular EC. Also, HUVEC and microvascular lymphatic endothelial cells have different expression levels for many molecules including VEGFR-3 [38], CD31, and VE-cadherin [39]. Because the new model of direct resting CD4+ T cell infection is based in a lymphoid context, studying T cell communication with microvascular EC is of higher in vivo relevance. Given that the study of communication between T cells and EC in the context of HIV latency has previously relied on macrovascular EC models, which are known to differ from more relevant microvascular EC models, in the present study we investigated the effects of microvascular EC (lymphatic EC) stimulation of resting CD4+ T cells in establishing HIV infection and latency.

## Methods

### Endothelial cells and in vitro infection assays

The two different types of endothelial cells used in this study were human lymphatic endothelial cells (LEC) and human umbilical vein endothelial cells (HUVEC or EC). LEC were purchased from ScienCell Research Laboratories (isolated from human lymph nodes) and cultured in media consisting of basal endothelial cell medium combined with 5% fetal bovine serum (FBS and 1% penicillin/streptomycin solution (P/S)). EC were purchased from PromoCell (Germany) and cultured in M199 media supplemented with 20% FBS and 1% P/S. Lymphatic endothelial cell growth factors (ScienCell) were added to LEC and endothelial cell growth factors (BD Biosciences) were added to EC fresh every 3 days to a final concentration of 50 μg/mL. When indicated, both types of endothelial cells were pre-treated with IFN-γ (50 ng/mL) (Invitrogen) for 3 days prior to the addition of resting T cells, which induced the expression of MHC class II. Endothelial cells were plated to 100% confluence and 300,000 resting T cells were co-cultured with LEC/EC per well of a 24-well plate, or up to 5 million T cells per well in a 6-well plate. Resting T cells were co-cultured with LEC/EC for 1 day in RPMI 1640 + 10% FBS + 1% Pen/Strep antibiotics (without LEC/EC growth factor or IFN-γ) prior to overnight infection. The co-cultures were maintained in the same media for the duration of the experiments. Expressions of GFP and T cell activation markers were examined on various days post infection using flow cytometry. Antibodies for various activation markers and CD58-PE were all purchased from BD Biosciences, and used according to manufacturer’s recommendations. For experiments on latent infections, flow cytometric sorting was also done at various days post infection. After sorting, the GFP- cells were cultured with or without PMA (10 ng/mL) plus Ionomycin (1 μg/mL) (both from Sigma) and Raltegravir (3.3 μM) (Selleck) for 2 days before flow cytometric analysis of GFP expression. In experiments involving activated T cells, PBMC were activated with phytohemagglutinin (PHA) and IL-2 (both 1 μg/mL) for 3 days prior to a negative bead depletion to isolate the CD4+ T cells. The activated T cells were co-cultured similarly to the resting T cells in the manner described above with addition of IL-2 (1 μg/mL) to the culture media.

### Virus production

The procedure for creating the GFP reporter virus has been previously described [31]. Briefly, the enhanced green fluorescence protein (eGFP or GFP) reporter virus was generated by cotransfecting HEK293T cells with a plasmid encoding NL43-dE-GFP and a plasmid encoding the HIV-1 envelope (pWE-CXCR4) using TrueFect (United Bio-systems) at a 2:1 ratio (pNL43:pWE). Supernatants were collected after 72 h and filtered through a 0.22 μm membrane to remove cell debris. Virus particles were pelleted using Lenti-X concentrator (Clontech Latoratories) by following the manufacturer’s instructions and resuspended with 1/27 of the original volume of RPMI + 10% FBS.

### Separation of various T cell populations

Human peripheral blood mononuclear cells (PBMC) were obtained from HIV- blood by centrifugation through a Ficoll-Hypaque density gradient at 300 x *g* for 40 min. Activated CD4+ T cells were purified from PBMC using Miltenyi microbeads (a negative depletion kit for isolating CD4+ T cells). Resting CD4+ T cells were purified from PBMC similarly with the addition of biotin labeled anti-CD25 and anti-HLA-DR antibodies to the Miltenyi depletion cocktail mix and subsequently increasing the amount of anti-biotin microbeads added. RO+/RA- memory T cells and RO−/RA+ naïve T cells were also purified using their respective Miltenyi negative depletion kits. Similarly, biotin labeled anti-CD25 and anti-HLA-DR antibodies were added, as were increased anti-biotin microbeads.

### Detection of latent infections

As described previously [31], in order to detect latent infection, infected T cells were sorted for GFP-negative cells on day 8 post-infection. GFP-negative cells were then cultured alone or activated with PMA and Ionomycin for 2 days along with the integrase inhibitor raltegravir to block any integration during activation. GFP expressions were then compared in cultures with or without activation.

### Detection of IL-6 using ELISA

Supernatants from cell culture wells were collected and frozen at − 80 °*C. ELISA* kits for IL-6 were purchased from BioLegend, and experiments were performed according to manufacturer’s instructions. 100 μL of supernatant was used from each sample in duplicates.

### Blocking IL-6 and CD2

Antibodies were used to block the effects of IL-6 and CD2 signaling in the resting T cell and LEC/EC co-cultures. When blocking IL-6, LEAF Purified anti-human IL-6 antibody (BioLegend) was added to the wells at various concentrations immediately after introducing resting CD4+ T cells to LEC/EC. For CD2 blocking, resting CD4+ T cells were incubated with LEAF Purified anti-human CD2 antibody (BioLegend) at various concentrations for 1 h prior to being co-cultured with LEC/EC. For both IL-6 and CD2 blocking, the antibody was refreshed 1 day post-infection. Infection levels were measured 6–8 days after infection.

### Ethical approval

This study was approved by the Internal Review Board (IRB) of Calvin College, reference number: 11–010.

## Results

### Kinetics of viral infection in resting CD4+ T cells co-cultured with lymphatic endothelial cells compared with HUVEC

**Fig. 1:**
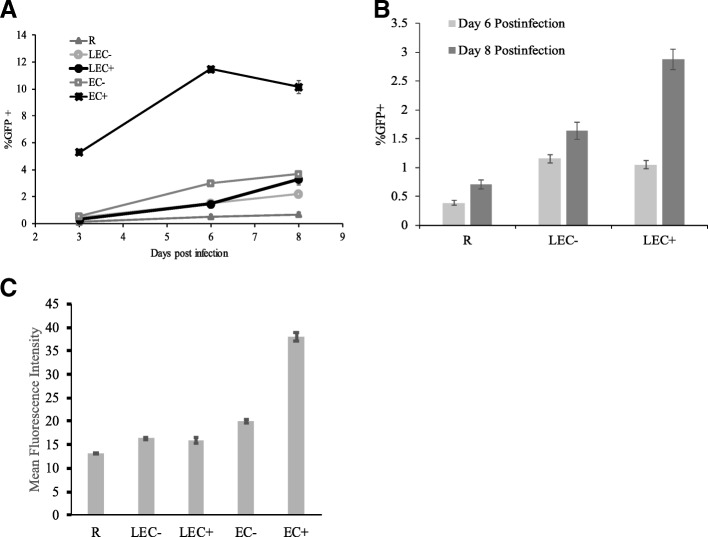
Kinetics of viral infection in resting CD4+ T cells co-cultured with LEC compared with HUVEC. Resting T cells were cultured alone, with human umbilical vein endothelial cells (EC), or human lymphatic endothelial cells (LEC). + and - indicate treatment with or without IFN-γ respectively in EC or LEC. All T cells were infected with an HIV reporter virus expressing GFP 1 day after co-culture, and the %GFP+ cells were measured **a** on days 3, 6, and 8 post-infection. Infection rates were substantially higher in LEC-stimulated resting T cells than in resting T cells alone (statistically significant, Student’s T-tests, *p* values 1.6 × 10^− 6^, 1.0 × 10^− 7^, and 7.8 × 10^− 5^ on days 3, 6, and 8, respectively) **b** A comparison of infection levels in LEC+ and LEC- stimulated T cells on day 6 and 8 post-infection. Samples were taken in triplicates and means+/− standard errors are plotted. Data shown are representative of seven independent experiments yielding similar results. **c** Mean fluorescence intensity data for GFP+ cells taken on day 8 post-infection. Samples were taken in triplicates and means+/− standard errors are plotted. Data shown are representative of seven independent experiments yielding similar results

Infection in resting CD4+ T cells stimulated by endothelial cells (HUVEC) takes place much slower than in activated T cells [31]. As we began investigating the effect of lymphatic endothelial cells (LEC) on resting T cells, we compared viral infection kinetics in LEC-stimulated resting T cells with those in HUVEC-stimulated T cells. Resting CD4+ T cells were isolated from HIV-negative donors and co-cultured with HUVEC (EC+ and EC-), LEC+ and LEC- (previously treated with or without IFN- γ respectively). Treatment of IFN- γ for 3 days induced expression of MHC II on LEC, similar to HUVEC. After 1 day of co-culturing, T cells were infected with the GFP reporter virus, and infection rates (% GFP+) were examined on various days post-infection. As shown in Fig. 1a, viral infection in HUVEC-stimulated resting T cells plateaued or even slightly decreased after day 6 (as was seen previously [31]), but for LEC-stimulated resting T cells, infection rates often continued to go up after day 6, especially for cells stimulated by LEC+. The proportion of GFP-expressing cells on any given day post infection was likely the combination of cell death in some infected cells (decrease in GFP+) and the emergence of new GFP-expressing cells (increase of GFP+). Here we gated on the live cells (higher forward scatter and lower side scatter) while assessing the level of GFP. It represents the proportion of live GFP producing cells at that moment, not the accumulative proportion of infected cells. Even though the overall infection rates in LEC-stimulated resting T cells were lower than the infection rates in HUVEC-stimulated T cells, on each of the 3 days measured, infection rates were substantially higher in LEC-stimulated resting T cells than in resting T cells alone (statistically significant, Student’s T-tests, *p* values 1.6 × 10^− 6^, 1.0 × 10^− 7^, and 7.8 × 10^− 5^ on days 3, 6, and 8, respectively, LEC- vs. R). Moreover, similar to HUVEC stimulation, LEC- stimulation resulted in increased infection in resting T cells, at similar levels or slightly lower than those in T cells stimulated by LEC+. Interestingly, while EC+ stimulation always resulted in higher infection rates than EC- stimulation [31], sometimes LEC- stimulation would result in higher infection rates than stimulation by LEC+, particularly on day 6 or earlier post infection (Fig. 1b). On day 8 post infection, LEC+ stimulation seemed to result in higher infection rates than LEC- (Fig. 1b). The increase of infection rates from day 6 to day 8 for LEC+ stimulated T cells can be two-fold or more in some donors (Fig. 1b). In Fig. 1c, mean fluorescence intensity data of the infected cells from day 8 post infection are shown. EC+ induced the most increase compared with R, though EC-, LEC−/+ all had small but statistically significant increase (Student’s T-tests, *p* values 3.9 × 10–8, 7.6 × 10–5, 0.004, 0.03 respectively). It was not surprising to see with EC+ stimulation the GFP intensities were higher because a small proportion of the resting T cells were activated by EC+.

### Resting T cells stimulated by LEC can be productively infected by HIV while remaining in resting state

**Fig. 2:**
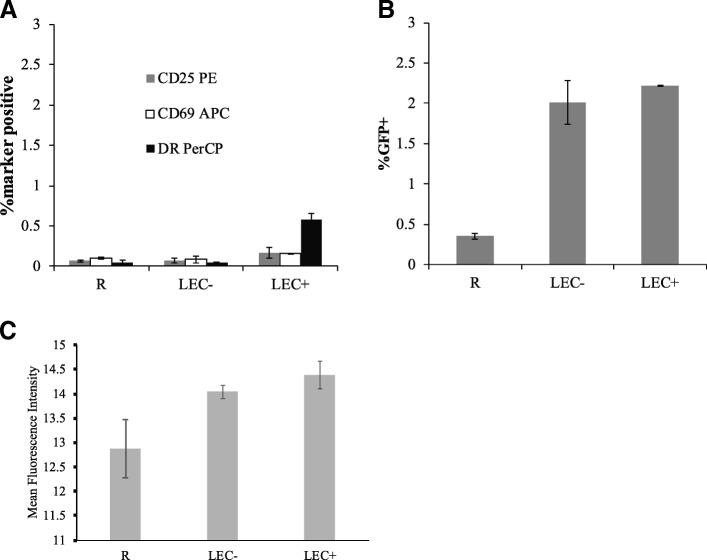
Resting T cells stimulated by LEC can be productively infected while remaining in resting state. Resting T cells were cultured alone or with human lymphatic endothelial cells (LEC). + and - indicate treatment with or without IFN-γ respectively in LEC. All T cells were infected with an HIV reporter virus expressing GFP 1 day after co-culture. On day 6 post-infection, T cells were stained to test for the presence of three activation markers: CD25-PE, CD69-APC, and DR-PerCP. Levels of activation are shown in (**a**) and levels of infection (%GFP+) are shown in (**b**). Samples were taken in triplicates and means +/− standard errors are plotted. Data shown are representative of three independent experiments yielding similar results. **c** Mean fluorescence intensity of the GFP+ cells, measured on day 6 post-infection, is shown. Samples were taken in triplicates and means +/− standard errors are plotted. Data shown are representative of three independent experiments yielding similar results

From our last study, we knew resting CD4+ T cells stimulated by HUVEC remain in a resting state while being infected [31]. To examine whether LEC stimulation would activate resting T cells, we measured cell activation markers CD25, CD69, and HLA-DR in LEC stimulated resting T cells on day 6 post infection. As shown in Fig. 2a, less than 0.6% of the T cells co-cultured with either LEC- or LEC+ expressed activation markers. In T cells co-cultured with LEC+, there were typically slightly more cells expressing activation markers than those cultured alone or with LEC-. These cells may recognize allogeneic MHC class II on LEC+ and become activated. A similar phenomenon was observed with HUVEC stimulation as well. However, the proportion of T cells that were infected was always significantly higher than the proportion of cells that were activated (compare Fig. 2a and b). Mean fluorescence intensity data for the infected T cells analyzed in Fig. 2b can be seen in Fig. 2c. There were slight increases of GFP intensities in LEC stimulated T cells, but they were not statistically significant.

### Resting memory T cells are preferentially infected compared to naïve T cells when co-cultured with LEC, but the pattern differs from HUVEC

In our previous study, we found that although naïve T cells co-cultured with HUVEC still showed greater infectivity than naïve T cells cultured alone, memory T cells were infected at much higher rates than naïve T cells in EC-stimulated cultures (both EC+ and EC-). This suggested that signals provided by EC to memory T cells were able to overcome the restrictions to a much greater extent than in naïve cells. This is consistent with the fact that EC express CD58 but not the co-stimulatory molecules CD80/86 and thus are better at stimulating memory T cells than naïve T cells. Naïve T cells generally require a stronger co-stimulatory signal (e.g. through CD80/86) for activation than memory T cells (reviewed in [37]).

**Fig. 3:**
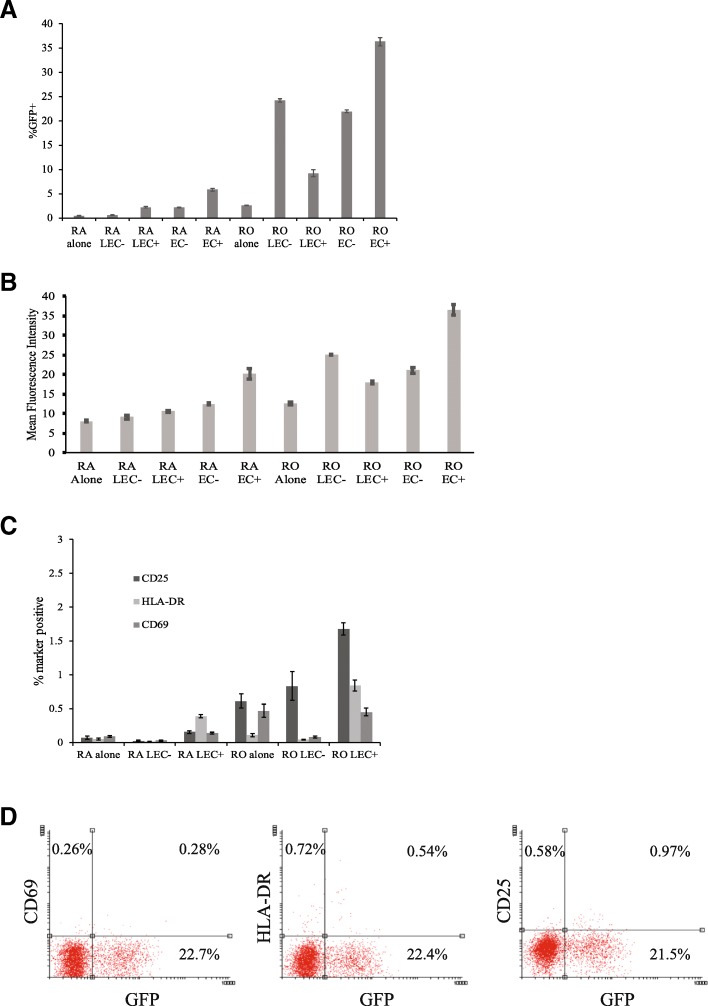
Resting memory T cells are preferentially infected than naïve T cells when co-cultured with LEC. **a** Infection in naïve and memory T cells stimulated with LEC and HUVEC (EC). Naïve resting T cells and memory resting T cells were each cultured alone, with human umbilical vein endothelial cells (EC), or human lymphatic endothelial cells (LEC). + and - indicate treatment with or without IFN-γ respectively in EC or LEC. All T cells were infected with an HIV reporter virus expressing GFP 1 day after co-culture, and the %GFP+ cells were measured on day 6 post-infection. Samples were taken in triplicates and means+/− standard errors are plotted. Data shown are representative of eight independent experiments yielding similar results. **b** Mean fluorescence data of the GFP+ cells in the experiment described in (A) are presented. Mean fluorescence intensity data were measured on day 6 post-infection. Samples were taken in triplicates and means+/− standard errors are plotted. Data shown are representative of eight independent experiments yielding similar results. **c** Activation in naïve and memory T cells stimulated by LEC. On day 6 post-infection, activation levels were examined in T cells by measuring the presence of three activation markers: CD25, HLA-DR, and CD69. **d** Same experiment as in (**c**). CD25, HLA-DR, and CD69 levels were individually plotted against GFP expression. Samples were taken in triplicates and means +/− standard errors are plotted. Data shown are representative of four independent experiments yielding similar results

In our current study, we examined the effects of LEC stimulation on the infection of resting memory and naïve T cells. Interestingly, we noticed a difference in the pattern of infection of memory cells co-cultured with LEC as opposed to HUVEC. As shown in Fig. 3a, we found that memory T cells stimulated by LEC- showed higher rates of infection than memory T cells stimulated by LEC+, suggesting that LEC stimulation of memory T cells is not dependent on interactions between MHC II and TCR. By contrast, in HUVEC stimulation, memory T cells stimulated by EC+ showed greater infection rates than memory T cells stimulated by EC- (Fig. 3a and [31]). For naïve T cells, we observed that LEC+ stimulation led to greater infection rates than LEC- stimulation which was similar to the pattern observed in HUVEC stimulation (Fig. 3a). Also similar to HUVEC, we observed that memory T cells showed greater infection rates than naïve T cells when co-cultured with both LEC+ and LEC- (Fig. 3a). The same trends observed in the analysis of GFP expression (Fig. 3a) are reflected in the mean fluorescence intensity data of infected cells presented in Fig. 3b.

### Resting memory T cells remain in resting phenotype after LEC stimulation

Our previous study has demonstrated that the majority of the resting T cells co-cultured with EC remain in a resting state throughout the course of infection [31].To similarly ensure that memory T cells were not activated after LEC stimulation, we measured the expression levels of activation markers (CD25, HLA-DR and CD69) in resting memory (and naïve) T cells after LEC stimulation. As seen in Fig. 3a, while the proportion of memory cells expressing activation markers was higher in LEC+ stimulated T cells than unstimulated T cells (in both RO and RA cells), it still accounted for a small percentage of the total population of memory cells. When GFP expressions (representing infection rates) were plotted against individual activation markers CD25, HLA-DR and CD69 for LEC+ stimulated memory T cells, one can see that while a small portion of the infected memory cells were positive for each activation marker tested, the majority of cells expressing GFP remained in a resting state during the course of infection (Fig. 3d).

### Latent viral infection in resting T cells co-cultured with LEC

**Fig. 4:**
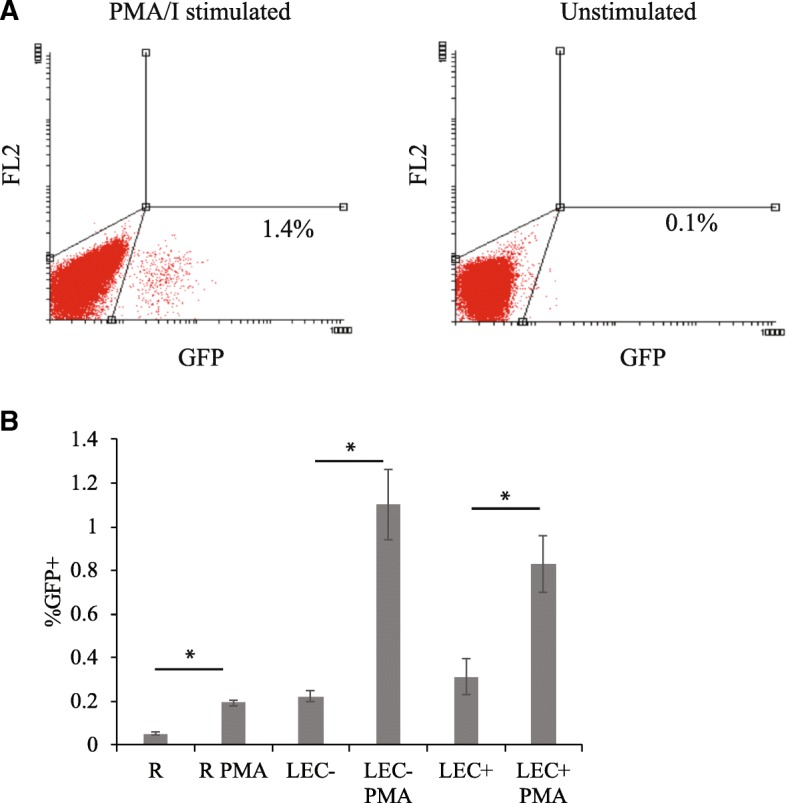
Latent viral infection in CD4+ resting T cells co-cultured with human lymphatic endothelial cells. Resting CD4+ T cells were cultured alone or with human lymphatic endothelial cells (LEC). + and - indicate treatment with or without IFN-γ respectively in LEC. All T cells were infected with an HIV reporter virus expressing GFP 1 day after co-culture. On day 8 post-infection, GFP negative cells were sorted and cultured with or without PMA/Ionomycin and raltegravir for 2 days to reactivate latent virus while preventing de novo infection. Following 2 days of reactivation, %GFP+ cells were measured. **a** GFP expression levels in PMA/I stimulated and unstimulated resting T cells after sorting from LEC- co-cultures. **b** GFP expression comparisons in resting T cell alone, LEC+ and LEC- co-cultures with and without PMA/I stimulation. *P* values from Student’s T tests comparing PMA/I stimulated cells to their unstimulated counterparts were 6 × 10^− 6^, 0.02, and 0.01, respectively. Data shown are representative of three independent experiments yielding similar results. Samples were taken in quadruplicates and means+/− standard errors are plotted. *Student t-test; *p* < 0.05

From our last study, we knew HUVEC-stimulated resting CD4+ T cells harbor latent infection [31]. To examine whether LEC stimulation would result in latent infection, we followed a similar procedure as was used with HUVEC. Resting CD4+ T cells were first co-cultured with LEC and then infected just as in productive infection experiments. On day 8 or 9 post-infection, after most unintegrated viral DNA had decayed [14] and most integrated virus had expressed GFP, GFP negative T cells were sorted out and activated with PMA and ionomycin (PMA/I) for 2 days. PMA/I is known to reactivate latent HIV, and integrase inhibitor raltegravir was also included in the cultures to prevent de novo viral integration during the 2 day culturing. Because some cells express GFP very slowly, a small amount of cells that were GFP negative at the time of sorting began to express GFP without stimulation over the next 2 days. As shown in Fig. 4a, there was some GFP expression in cells without PMA/I stimulation, but there was an increase of GFP expression after PMA/I stimulation, demonstrating the expression of latent virus upon activation of T cells. The increase of GFP expression after PMA/I stimulation was shown in resting T cells alone and resting T cells co-cultured with either LEC- or LEC+ (Fig. 4b). The increase was most numerically dramatic for the LEC- co-culture (Student’s T test, *p* value 0.01) but was also statistically significant for the resting alone (R) (Student’s T test, p value 6 × 10^− 6^) and LEC+ cultures (Student’s T test, p value 0.02). These results signify that LEC stimulation results in post-integration latent infection in resting T cells.

### IL-6 is involved in the interaction between LEC and resting CD4+ T cells

**Fig. 5:**
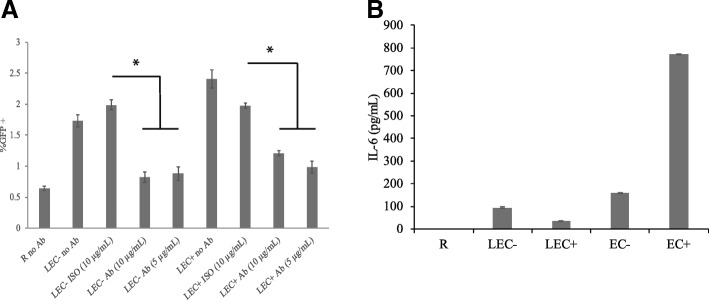
IL-6 is involved in interactions between human lymphatic endothelial cells and resting CD4+ T cells. **a** Resting CD4+ T cells were cultured alone or with human lymphatic endothelial cells (LEC). + and - indicate treatment with or without IFN-γ respectively in LEC. At the time of co-culture, anti-human IL-6 antibody was added at various concentrations (5 and 10 μg/mL). Isotype control antibodies were also included as a negative control. All T cells were infected with an HIV reporter virus expressing GFP 1 day after co-culture. %GFP+ cells were measured on day 7 post-infection. Data shown are representative of three independent experiments yielding similar results. Samples were taken in triplicates and means+/− standard errors are plotted. *Student t-test; *p* < 0.05. **b** Differential IL-6 levels in HUVEC-stimulated and LEC-stimulated resting T cells. Resting T cells were cultured alone, with human umbilical vein endothelial cells (EC), or human lymphatic endothelial cells (LEC). + and - indicate treatment with or without IFN-γ respectively in EC or LEC. All T cells were infected with an HIV reporter virus expressing GFP 1 day after co-culture, and supernatants were taken for ELISA analysis on day 6 post-infection. Samples were taken in triplicates and means+/− standard errors are plotted. Data shown are representative of four independent experiments yielding similar results

The pro-inflammatory cytokine IL-6 was found to be produced by HUVEC and was involved in HUVEC stimulation of resting T cells [32]. To examine whether IL-6 was involved in LEC stimulation as well, we decided to block it with an anti-IL-6 antibody in LEC-T cell co-cultures. LEC were plated for at least 3 h before resting CD4+ T cells were added to the LEC after supernatants were removed from plated LEC. At the same time, an anti-human IL-6 antibody was added to the co-culture at various concentrations (5 and 10 μg/mL). Isotype control antibodies were also included as a negative control. After 1 day, T cells stimulated by LEC, with or without anti-IL-6 antibody, were infected, and GFP levels were measured on day 6 or 7 post-infection. As seen in Fig. 5a, the addition of anti-IL-6 antibody resulted in significantly lower infection rates in resting cells stimulated by LEC- and LEC+. In the case of resting cells stimulated by LEC-, the addition of anti-IL-6 antibody nearly reduced the infection rates to the level of unstimulated resting cells, suggesting that IL-6 was almost solely responsible for the effect of LEC- stimulation. For LEC+ stimulation, however, addition of anti-IL-6 antibody did not completely reduce the infection level to that of unstimulated resting cells, suggesting that for LEC+ cells, factors other than IL-6 were also involved in stimulation of resting T cells.

### IL-6 levels are lower in LEC-stimulated T cells than EC-stimulated T cells

Once we knew IL-6 is involved in EC and LEC stimulation of resting CD4+ T cells, we set out to measure IL-6 levels at the end of infection (day 6 post infection) in T cells co-cultured with EC or LEC using ELISA, to see if IL-6 levels correlated with infection levels. We found that EC co-cultures had more IL-6 than LEC co-cultures in general (Fig. 5b). This correlates with what we know of their respective infection rates (Fig. 1a). More interestingly, while EC+ co-cultures generally had more IL-6 than EC- ones (corresponding to infection rates), LEC- co-cultures had more IL-6 than LEC+ co-cultures (Fig. 5b). This may indicate that in T cells stimulated by LEC+, other factors in addition to IL-6 are playing a role in infection, which is consistent with our findings in IL-6 blocking experiments (Fig. 5a).

### Involvement of CD2 signaling in EC and LEC stimulation of resting CD4+ T cells

**Fig. 6:**
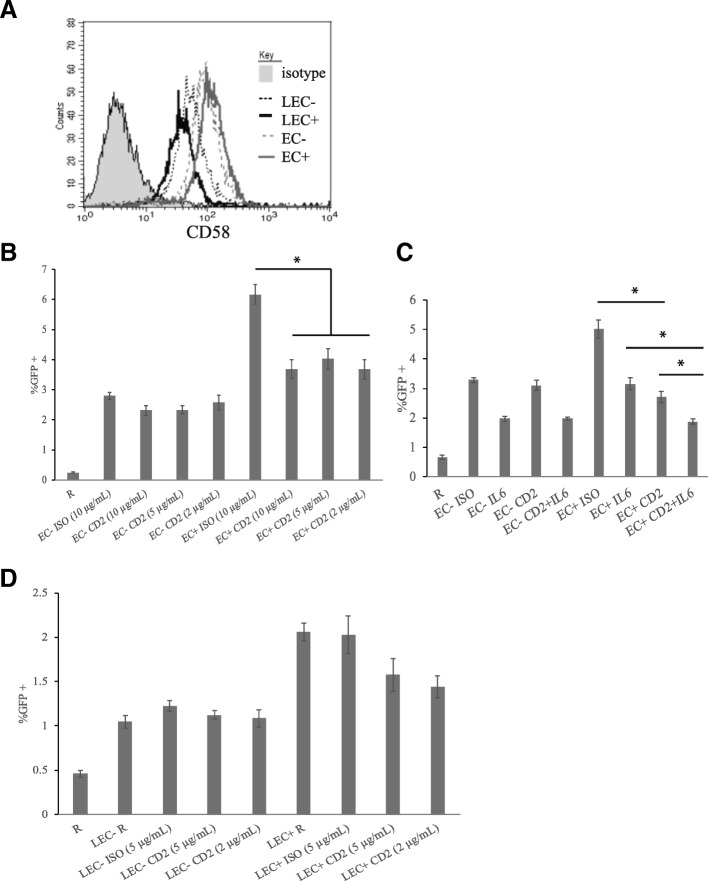
Differential involvement of CD2 in HUVEC and LEC stimulation of resting T cells. **a** CD58 expression in HUVEC (EC) and LEC. EC and LEC were stimulated with or without IFN-γ for 3 days (+/− respectively), and CD58 expressions were measured. **b** CD2 involvement in HUVEC stimulation of resting CD4+ T cells. Resting CD4+ T cells were cultured alone as a control or with human umbilical vein endothelial cells (EC). One hour before co-culturing with LEC, CD2 blocking antibodies were added at various concentrations (2, 5, and 10 μg/mL) to resting CD4+ T cells. Isotype control antibodies were also included as a negative control. All T cells were infected with an HIV reporter virus expressing GFP 1 day after co-culture. %GFP+ cells were measured on day 6 post-infection. Samples were taken in triplicates and means+/− standard errors are plotted. Data shown are representative of four independent experiments yielding similar results. *Student t-test; *p* < 0.05 **c** Similar to **b**, with both anti-CD2 and anti-IL-6 antibodies. One hour before co-culture, CD2 blocking antibodies (2 μg/mL), IL-6 blocking antibodies (5 μg/mL), and a combination of CD2 blocking antibodies and IL-6 blocking antibodies were added. Isotype control antibodies were also included as a negative control. **d** CD2 involvement in LEC stimulation of resting CD4+ T cells. Resting T cells were cultured alone, with human lymphatic endothelial cells (LEC). One hour before co-culture, CD2 blocking antibodies, IL-6 blocking antibodies, and a combination of CD2 blocking antibodies and IL-6 blocking antibodies were added at indicated concentrations. Isotype control antibodies were also included as a negative control. All T cells were infected with an HIV reporter virus expressing GFP 1 day after co-culture. %GFP+ cells were measured on day 6 or 7 post-infection. Samples were taken in triplicates and means+/− standard errors are plotted. Data shown are representative of five independent experiments yielding similar results. *Student t-test; *p* < 0.05

Originally, Choi et al. found CD2-CD58 interaction to be involved in EC stimulation of resting CD4+ T cells. ECs express CD58, which is a known co-stimulatory molecule that binds to CD2 on T cells, and so do LECs (Fig. 6a). We used various concentrations (2, 5 and 10 μg/mL) of CD2 blocking antibodies to assess the involvement of CD2 signaling in EC stimulation of T cells. We found that infection in EC+ stimulated T cells was significantly blocked (by about 50%) at all antibody concentrations, while infection in EC- stimulated T cells was largely unaffected (Fig. 6b). When anti-IL-6 antibody was combined with anti-CD2 antibody, there was added blocking effect for infection in EC+ stimulated T cells, but for EC- stimulated T cells, there was no difference between anti-IL-6 antibody alone and with both antibodies (Fig. 6c). This further demonstrated the lack of involvement of CD2 in EC- stimulation of resting T cells. We then examined whether CD2 signaling is involved in LEC stimulation of resting T cells. For LEC- stimulated T cells, similar to EC- stimulated T cells, blocking CD2 had no effect on infection rates; whereas for LEC+ stimulated T cells, unlike EC+ stimulated T cells, there were consistently no effects or just a slight decrease (not statistically significant) in infection rates with CD2 antibodies (Fig. 6d).

### LEC stimulation increases infection rates in activated CD4+ T cells

**Fig. 7:**
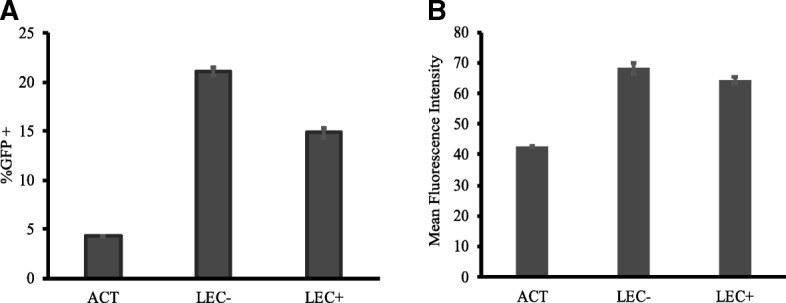
LEC stimulation increases infection rates in activated CD4+ T cells. PBMC were activated with PHA (1 μg/mL) for 3 days before isolating CD4+ T cells by bead depletion. The activated T cells were then cultured alone, with LEC-, or LEC+. All T cells were infected with an HIV reporter virus expressing GFP 1 day after co-culture. **a** %GFP+ cells were measured 3 days after infection. Samples were taken in triplicates and means+/− standard errors are plotted. Data shown are representative of four independent experiments yielding similar results. Student’s T tests showed significant differences between cells by stimulation. ACT-LEC-: *p* = 9.99 × 10^− 11^, ACT-LEC+: *p* = 6.40 × 10^− 9^, LEC + -LEC-: 5.36 × 10^− 8^. **b** Same experiment as (A). Mean fluorescence intensities of GFP+ cells are shown. Samples were taken in triplicates and means+/− standard errors are plotted. Data shown are representative of four independent experiments yielding similar results. Student’s T tests showed significant differences between cells by stimulation. ACT-LEC-: *p* = 7.26 × 10^− 8^, ACT-LEC+: *p* = 7.71 × 10^− 9^, LEC + -LEC-: .0015

There are some evidences that activated CD4+ T cells may also play a role in HIV persistence [40], and a study showed that mucosal stromal fibroblasts increased HIV infection rates in activated T cells [28]. Therefore, we sought to investigate the effects of LEC stimulation on the infection of activated CD4+ T cells. We activated donor PBMC with PHA and IL-2 for 3 days before isolating total CD4+ T cells. We then co-cultured the activated CD4+ T cells alone (ACT), with LEC+, or with LEC- and observed the corresponding infection rates 3 days post infection. We found that LEC stimulation dramatically increased HIV infection rates in the activated T cells, with T cells stimulated by LEC- showing the highest infection rates followed by those stimulated by LEC+ and those left unstimulated (Fig. 7a). Significant differences were observed between all stimulation conditions; ACT-LEC-: *p* = 1 × 10^− 10^, ACT-LEC+: *p* = 6.4 × 10^− 9^, LEC + -LEC-: 5.36 × 10^− 8^. A similar trend was observed was observed when analyzing the mean fluorescence intensity of the cells in each treatment group; cells stimulated by LEC- had the highest mean fluorescence intensity followed by LEC + −stimulated cells and unstimulated cells (Fig. 7b). ACT-LEC-: *p* = 7.26 × 10^− 8^, ACT-LEC+: *p* = 7.71 × 10^− 9^, LEC + -LEC-: 0.0015.

## Discussion

In this study, we demonstrated that LEC stimulation could promote direct HIV infection of resting CD4+ T cells, just as HUVEC stimulation did ([31, 32]). We were able to confirm that LEC stimulation rendered resting CD4+ T cells much more prone to infection than these T cells alone (Fig. 1). Most importantly, LEC stimulation also increased latent infection in resting T cells (Fig. 4), similar to HUVEC stimulation, which speaks to the importance of the involvement of endothelial cells in HIV persistence.

Other similarities between LEC stimulations and HUVEC stimulations include the following: the stimulated T cells remained largely un-activated (Fig. 2 and Fig. 4b) and showed slower infection kinetics than infection in activated T cells (Fig. 1a); among EC-stimulated T cells, memory T cells were preferentially infected, even though naïve T cells also had increased infection rates compared with un-stimulated T cells (Fig. 3a); and IL-6 is involved in the interaction between T cells and endothelial cells (Fig. 5a).

However, we also found significant differences in HIV infection of LEC-stimulated T cells compared with EC-stimulated T cells. Previous studies utilizing macrovascular endothelial cells have maintained that to achieve highest infection levels, EC stimulation of resting T cells requires that EC express MHC II [26, 27]. Indeed, for HUVEC stimulation in our studies, EC+ always induced more infection than EC- in any type of T cells, whether memory or naive. Strikingly, we observed that memory T cells stimulated by LEC- showed higher rates of infection than memory T cells stimulated by LEC+ (Fig. 3a). In contrast to HUVEC stimulation, this indicates that LEC stimulation of memory T cells is not dependent on MHC II – TCR interactions. The interactions between MHC II on the EC+/LEC+ and TCR on the T cells are slightly artificial in these in vitro studies, since EC+/LEC+ used in our experiments were not from the same donor as the T cells; hence there were low levels of mixed lymphocyte reactions. In vivo, T cells do not respond to self MHC, thus there is no mixed lymphocyte reaction involved. Since EC-/LEC- do not express MHC II, and there were no mixed lymphocyte reactions involved, the fact that LEC- promotes high level of HIV infection in resting T cells suggests that MHC II – TCR interactions are not required in LEC stimulation of T cells, which may have more in vivo relevance. In addition, we were very intrigued by the finding that LEC could induce significant infection in *memory* CD4+ T cells, both productively and in latent infection. Memory T cells, as opposed to naïve T cells, are the majority of CD4+ T cells harboring latent reservoir in vivo, and LEC stimulation may provide a mechanism for latent reservoir formation in vivo*.*

Our results suggest that IL-6 is involved in the interaction between LEC- and T cells (Fig. 5a). However, IL-6 alone does not induce as high of an infection level as EC- or LEC- ([32] and unpublished data). This suggests that IL-6 may be necessary but not sufficient in inducing high level of infection, or there are additional factors involved in the stimulation. Further studies are required to investigate the involvement of other factors in addition to IL-6. It was also interesting to discover that LEC co-cultures had much less IL-6 than EC co-cultures, even though IL-6 was clearly involved in LEC stimulation of resting CD4+ T cells (Fig. 5b). This again highlighted the importance of investigating resting T cell stimulation by microvascular endothelial cells as this interaction has higher in vivo relevance than stimulation by macrovascular endothelial cells.

Another difference we observed was the lack of involvement of CD2 in interactions between LEC and T cells (Fig. 6d), given that CD2 was involved in EC+ interactions with T cells (Fig. 6b and c, and [30]). Since LEC also express CD58 (Fig. 6a), we wondered why LEC+ did not stimulate T cells similarly to EC+. As a matter of fact, EC- and LEC- both express comparable levels of CD58 to EC+ and LEC+, but anti-CD2 blocking antibody had no effect on their stimulations of T cells. It is possible that in EC-, LEC-, and LEC+ stimulations of resting T cells there were redundant factors involved, so blocking CD2 did not affect infection rates; whereas in EC+ cells, the redundant factor was absent, and CD2 was the only molecule involved in cell-cell contact, thus blocking CD2 had an effect. It is also possible, and probably more likely, that two molecules were needed in stimulation by EC+: CD2 and another factor; thus, blocking CD2 would reduce the infection rates. Because EC-, LEC- and LEC+ lacked the other factor they therefore all induced lower infection rates than EC+, and blocking CD2 had no effect since CD2 alone could not induce more infection. Either way, this again highlighted the fact that there were differences between HUVEC and LEC, and further studies need to be carried out to investigate the interactions between CD4+ T cells and LEC.

We also demonstrated that LEC stimulation promotes infection in activated CD4+ T cells (Fig. 7). This finding is relevant given the growing evidences that cell-cell interactions are important in HIV infection, and the recognition of the role of activated CD4+ T cells play in HIV infection and latency. Our results indicate the significance of studying the intercellular interactions of both resting and activated CD4+ T cells in the lymphoid context.

It should be noted that there are several limitations to our study. Firstly, while our novel model of LEC stimulation of resting CD4+ T cells during HIV infection is more physiologically relevant than the HUVEC model, an in vitro model is incapable of perfectly representing in vivo conditions. Also, although clear patterns emerged, because we used human primary cells, inherent donor to donor variations were observed in our experiments. In order to show the patterns clearly, for all the data in this paper we chose to utilize a single representative donor to illustrate the same patterns seen across multiple donors and experiments, rather than compiling data from all donors together. Finally, our study utilized a pseudotyped virus. While such a virus enabled us to investigate latent infection with GFP sorting, it may not represent in vivo strains perfectly. Although previous research has demonstrated that EC stimulation of CD4+ T cells promoted HIV infection with both a pseudotyped virus and a primary isolate [26], similar experiments need to be done with clinical isolates in the future to confirm our findings in LEC. In addition, while we were able to gain insight into certain interactions between LEC and the T cells, further studies are needed to decipher additional mechanisms allowing direct HIV infection of resting T cells upon LEC stimulation. We are investigating potential molecules and mechanisms through results from RNAseq experiments, where we compared gene expressions in resting T cells with and without stimulation by endothelial cells, and we hope to report positive findings soon.

## Conclusions

Overall, with this study, we confirmed that endothelial cells do promote HIV infection of resting CD4+ T cells, both productively and with latent infection, while keeping the T cells in a resting state. Our findings continue to highlight the significance of endothelial cells as well as the lymphoid tissue microenvironment in HIV persistence. We also observed significant distinctions between T cell stimulation by macrovascular EC (HUVEC) and microvascular lymphatic endothelial cells (LEC), most notably the lack of involvement of MHC II – TCR interactions in LEC stimulation of resting memory T cells. In this study, we demonstrated that microvascular lymphatic endothelial cells, which physiologically interact with T cells in vivo, could stimulate CD4+ resting T cells and promote HIV infection similarly to macrovascular HUVEC as previously observed. We also showed that LEC stimulation promotes HIV infection in activated CD4+ T cells. These conclusions provide insight into the model of direct infection of resting T cells and open doors for further investigation into the role of interactions between endothelial cells and both resting and activated T cells in HIV infection and persistence.

## Abbreviations

CD2: Cluster of differentiation 2
CD25: Cluster of differentiation 25
CD31: Cluster of differentiation 31
CD4: Cluster of differentiation 4
CD58: Cluster of differentiation 58
CD69: Cluster of differentiation 69
cDNA: Complementary DNA
EC +: Human umbilical vein endothelial cells treated with IFN-γ
EC: Endothelial cells (general), specifically human umbilical vein endothelial cells where indicated
EC-: Human umbilical vein endothelial cells not treated with IFN-γ
ELISA: Enzyme-linked immunosorbent assay
FBS: Fetal bovine serum
GFP: Green fluorescent protein
HIV: Human immunodeficiency virus
HLA-DR: Human leukocyte antigen – antigen D related
HUVEC: Human umbilical vein endothelial cells
IFN-γ: Interferon gamma
IL-2: Interleukin-2
IL-6: Interleukin-6
LEC +: Lymphatic endothelial cells treated with IFN-γ.
LEC: Lymphatic endothelial cells
LEC-: Lymphatic endothelial cells not treated with IFN-γ
MHC II: Major histocompatibility complex class II
P/S: Penicillin/Streptomycin
PBMC: Peripheral blood mononuclear cells
PHA: Phytohemagglutinin
PMA: Phorbol myristate acetate
PMA/I: Phorbol myristate acetate / Ionomycin
RA: CD45RA
RO: CD45RO
RPMI: Rosewell Park Memorial Institute medium
TCR: T-cell receptor
VEGF: Vascular endothelial growth factor
